# Two cases of multinodular and vacuolating neuronal tumour

**DOI:** 10.1186/2051-5960-2-7

**Published:** 2014-01-20

**Authors:** Istvan Bodi, Olimpia Curran, Richard Selway, Robert Elwes, Juan Burrone, Ross Laxton, Safa Al-Sarraj, Mrinalini Honavar

**Affiliations:** 1 Department of Clinical Neuropathology, King’s College Hospital NHS Foundation Trust, London SE5 9RS, United Kingdom; 2 Department of Neurosurgery, King’s College Hospital NHS Foundation Trust, London, United Kingdom; 3 Department of Neurophysiology, King’s College Hospital NHS Foundation Trust, London, United Kingdom; 4 MRC Centre for Developmental Neurobiology, King’s College London, London, United Kingdom; 5 MRC London Neurodegenerative Diseases Brain Bank, Institute of Psychiatry, King’s College, London, United Kingdom; 6 Servico de Anatomia Patologica, Hospital Pedro Hispano, Matosinhos, Portugal

**Keywords:** Dysembryoplastic neuroepithelial tumour, Ganglioglioma, Epilepsy, Brain tumour

## Abstract

An unusual multinodular and vacuolating neuronal tumour (MVNT) has been described in the cerebral hemispheres of ten patients with adult-onset seizures. We report the findings in two cases with similar features, a surgical resection and the other an autopsy specimen.

Case 1, a 34-year-old female, underwent surgical resection for a multinodular non-enhancing frontal white matter lesion causing intractable epilepsy. Case 2, presented with motor neurone disease (MND) at the age of 71 and MRI scanning revealed extensive multinodular non-enhancing white matter lesions in the temporal lobe. There was no history of epilepsy and post mortem histology confirmed MND.

Macroscopically multiple small grey well-formed, discrete and coalescent nodules were seen in the deep cortex and subcortical white matter. On histology, mature-looking neurons with large cytoplasmic vacuoles were distributed in a fibrillary background, where vacuoles were also noted. In the resected tumour scattered oligodendroglia-like cells were present. No ganglion cells were seen. The vacuolated cells exhibited immunopositivity for synaptophysin, HuC/HuD and p62 but were negative for NeuN, neurofilament, GFAP, IDH1, nestin and CD34. Electron microscopy showed non-membrane bound cytoplasmic vacuoles in the neurons and in some neuronal processes. The seizures recurred in Case 1.

Some clinicopathological features of this lesion suggest a possible relationship with dysembryoplastic neuroepithelial tumour (DNT) although the morphological features are not typical of DNT. Case 2 demonstrates that MVNT may remain asymptomatic.

## Background

The improving imaging techniques and the availability of surgery for the treatment of chronic, drug-resistant epilepsy have lead to the recognition of indolent brain tumours that appear to be largely restricted to this clinical setting. Many are of glio-neuronal lineage of which dysembryoplastic neuroepithelial tumour (DNT) and ganglioglioma are to be the most frequent [1]. However, there still appears to a lack of consensus on the criteria to diagnose these lesions, and there continue to be cases where the appearances do not conform to established criteria for diagnosis and may remain unclassifiable. Most epileptogenic neuroepithelial tumours present in childhood but may not get surgical treatment until adulthood due to their indolent clinical behaviour. Recently, a non-neurocytic, purely neuronal lesion affecting adults with epilepsy has been described, in which there was striking vacuolation of the neuronal cytoplasm, referred to as multinodular and vacuolating neuronal tumour (MVNT) [2]. We report two cases with similar histological features, one from a surgical resection to treat chronic seizures, and the other from an autopsy specimen without a history of epilepsy, with MRIs in both cases suggesting a diagnosis of DNT.

## Case presentation

### Case 1

**Figure 1:**
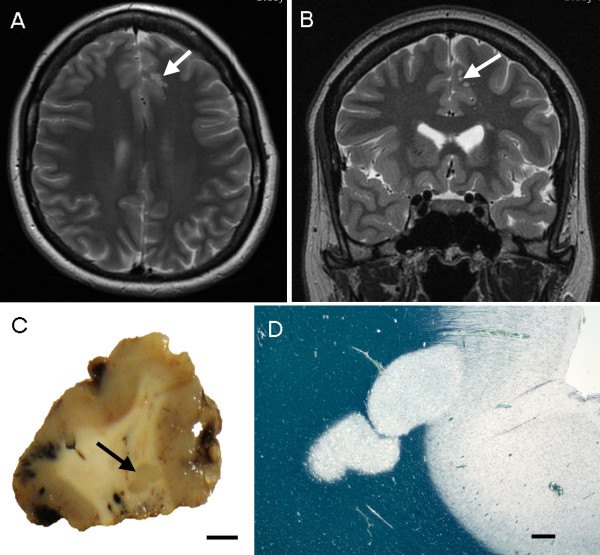
**Case 1.** Magnetic resonance imaging (MRI) and macroscopy. **(A-B)** T2-weighted MRI showing hyperintense, multinodular solid lesions in the subcortical white matter of the medial part of the left superior frontal gyrus without tissue expansion or mass effect (white arrow). **(C)** Coronally sectioned surgically removed material reveals coalescing grey nodules (black arrow) (bar = 5 mm). **(D)** Low power microscopy demonstrates hypomyelinated plaque-like nodules extending into the deep cortex (Luxol fast blue – Nissl) (bar = 1 mm).

A 34-year-old female, who suffered from intractable epilepsy since age 8, was found on MRI to have multiple small T2-weighted hyperintense lesions within the subcortical white matter of the antero-medial aspect of the left superior frontal gyrus (Figure 1), suggestive of DNT. There was a slight asymmetry of the hippocampi but no typical hippocampal sclerosis. There was no history of febrile convulsions in childhood. She had increasing frequency of nocturnal seizures, up to 30 a night, which were uncontrollable by various medications. She underwent a stealth-guided excision of the left frontal lesion in March 2010. Post-operatively, although the frequency of seizures decreased by 50% and the quality of her sleep improved, she was not seizure-free. In May 2012 she had elective admission for repeat electrode strip analysis and resection of the frontal lesion. Some seizure activity was recorded by the implanted electrodes, but the location was not consistent with a radiological abnormality or the patient’s seizure profile, and no further surgical resection was performed.

### Case 2

**Figure 2:**
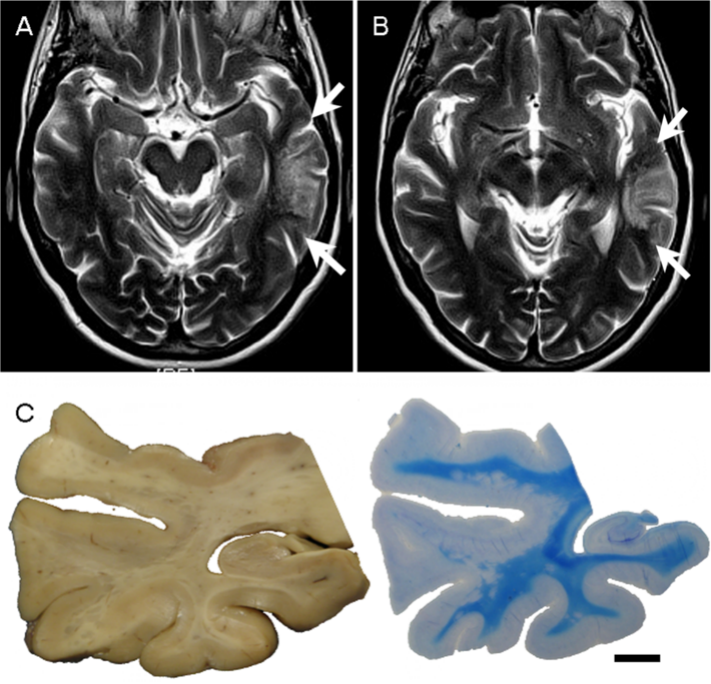
**Case 2.** MRI and macroscopy. **(A-B)** MRI showing extensive, T2-weighted hyperintense, nodular lesions in the white matter of the left posterior temporal lobe (between white arrows). No change was noted on follow up MRI in 15 months. **(C)** Coronally sectioned temporal lobe block from autopsy reveals numerous coalescing grey nodules along the junction of cortex and grey matter and in the subjacent white matter, which appear as hypomyelinated nodules by Luxol fast blue – Nissl (bar = 1 cm).

A 71-year-old female was diagnosed with a motor neuron disease (MND) with predominant bulbar involvement in July 2010 following one year history of dysarthria and increased difficulty with swallowing. She had no history of epilepsy or dementia. EMG was reported as consistent with anterior horn cell disease and treatment with Riluzole 50 mg was initiated. MRI brain scan in July 2010 showed a lesion with a high T2 signal in the left lateral temporal lobe (Figure 2), suggestive of low grade glioma or DNT. Due to the unusual appearances, she had another brain MRI in September 2011, which showed persistent signal change without enhancement, any restricted diffusion or any noticeable increase in size. She had a congenital cyst in her right eye and underwent a mastectomy for breast cancer in 2003. She passed away in May 2012 at the age of 73.

### Materials and methods

The surgically resected material (Case 1) was fixed in buffered formalin for 24–36 hours. All sampled tissue blocks were processed and embedded in paraffin wax and stained by conventional haematoxylin and eosin (H&E). Selected blocks showing histological abnormalities were further stained with Luxol-fast blue/Nissl (LFB/N) and PAS-D. Immunohistochemistry was carried out with antibodies against neuronal nuclear protein (NeuN), neuronal protein HuC/HuD (HuC/HuD), pan-neurofilament (pan-NF), neurofilament 200 KD (NF200 KD), synaptophysin, chromogranin, glial fibrillary acidic protein (GFAP), nestin, CD3, CD45, CD68, CD34, human leukocyte antigen-DR (HLA-DR), isocitrate dehydrogenase1 (IDH1) R132H, calbindin, parvalbumin, calretinin, Ankyrin-G, p62 and Ki67.

The brain and the spinal cord (Case 2) were consented to research and donated to the MRC London Neurodegenerative Disease Brain Bank at King’s College Hospital. After fixation in buffered formalin for 12 weeks, the whole brain and the spinal cord were sampled as per our departmental protocol for MND. All sampled blocks were stained with H&E and selected blocks were stained with LFB/N, p62, TDP-43, tau, CD34, CD68, PAS, Sudan black, Nestin, NeuN, pan-NF, NF200 KD, Ki67, CD68, HLA-DR, CD45, CD3, α-synuclein, amyloid precursor protein (APP).

Sections of 7 μm thickness (14 μm for LFB/N) were cut from paraffin-embedded blocks. Immuno-staining was performed using a Bond-max™ automated staining system (Leica Biosystems, Newcastle, United Kingdom). The immunolabelling was visualised using the Bond Polymer Refine Detection kit (DS9800, Leica Biosystems, Newcastle UK). The negative controls were treated identically except that primary antibody was omitted. The sources of the antibodies, and respective dilution and pre-treatment are described in Table 1.

**Table 1:** The sources of the antibodies used, and respective pre-treatment and dilution

**Antibody**	**Dilution**	**Pre-treatment**	**Supplier**	**Code**
Ankyrin-G	1:100	H2(20)	NeuroMab	75-146
APP	1:7500	H2(10)	Chemicon	*MAB348*
Calbindin	1:250	H2(20)	Sigma	*C7354*
Calretinin	1:100	H1(20)	Novocastra	*NCL-CALRETININ*
CD3	1:50	H2(30)	Dako	*M7254*
CD34	1:150	H2(20)	Dako	*M7165*
CD45 (LCA)	1:200	H1(30)	Dako	*M0701*
CD68 (PGM1)	1:50	H2(20)	Dako	*M0876*
Chromogranin	1:100	H2(20)	Novocastra	*NCL-CHROM-430*
GFAP	1:1000	H1(5)	Dako	*Z0334*
HLA-DR (cr3/43)	1:300	H2(20)	Dako	*M0775*
HuC/HuD	1:160	H1 (30)	Molecular probes	A-21271
IDH1	1:50	H1(20)	Dianova	*DIA H09 L*
Ki67	1:100	H2(20)	Dako	*M7240*
Nestin	1:200	H2(20)	Santa Cruz biotech	*sc-23927*
Neu N	1:2000	H1(20)	Chemicon	*MAB377*
NF (pan-NF)	1:100	H2(30)	Zymed	*18-0171Z*
NF200 KD	1:500	H1(5)	Dako	*M0762*
p62	1:100	H2(20)	BD Transduction labs	*610833*
Parvalbumin	1:500	H2(20)	Millipore (Chemicon)	*MAB1572*
Synaptophysin	1:40	H2(20)	Dako	*M0776*
Synuclein	1:500	Offline*	BD Transduction labs	*610787*
Tau (AT8)	1:500	H1(20)	Innogenetics	*MN1020*
TDP43-P	1:1500	H1(30)	Cosmo Bio Ltd.	*TIP-PTD-P02*

Pre-treatment codes: H1(5) = Epitope Retrieval Solution 1 (ER1) for 5 minutes; H1(10) = Epitope Retrieval Solution 1 (ER1) for 10 minutes; H2(20) = Epitope Retrieval Solution 2 (ER2) for 20 minutes; H2(30) = Epitope Retrieval Solution 1 (ER1) for 10 minutes. *Synuclein offline pre-treatment: 60 minutes in 80% Aqueous Formic Acid.

Electron microscopy was performed on material re-embedded from paraffin block in Case 1 and from formalin fixed wet tissue in Case 2 [3].

Genomic DNA was extracted from formalin fixed paraffin embededd tissue using the QIAamp DNA FFPE Tissue kit (Qiagen GmbH - Hilden, Germany). Bisulphite conversion was performed using an EpiTect kit (Qiagen GmbH - Hilden, Germany). MGMT promoter methylation status and BRAF V600 mutation were determined using the therascreen MGMT Pyro Kit and therascreen BRAF Pyro Kit respectively using a Q24 MDx Pyrosequencer (Qiagen GmbH - Hilden, Germany). Sequencing of IDH1 and IDH2 was also performed by pyrosequencing methods [4,5].

## Results

### Macroscopic findings

#### Case 1

The specimen consisted of 4 fragments measuring 30 × 30 × 20 mm, 20 × 10 × 5 mm, 20 × 10 × 3, and 15 × 10 × 3 mm. On slicing at 5 mm intervals, two grey nodules of 2 -3 mm were identified in the subcortical white matter (Figure 1C).

#### Case 2

The formalin fixed brain weight was 1198 g. Macroscopic examination of the brain revealed no significant cortical atrophy but the spinal cord showed mild atrophy of the anterior motor roots, consistent with MND. Coronal slices revealed widespread multiple discrete grey, plaque-like nodules measuring 1-3 mm scattered in the white matter of the posterior part of the left temporal lobe (Figure 2C).

### Microscopic findings

**Figure 3:**
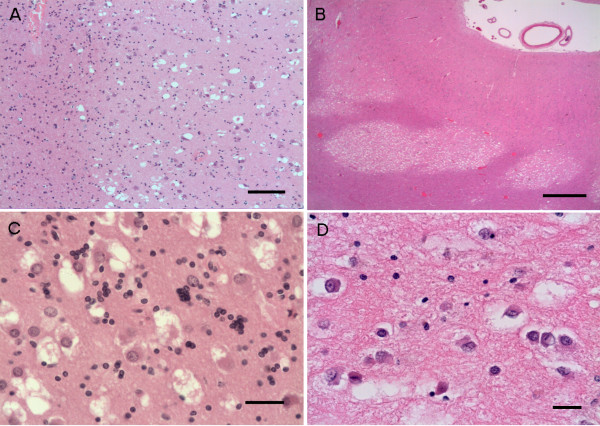
**Histology of Case 1 (A,C) and Case 2 (B,C). (A-B)** The nodular lesions are well demarcated in both cases. **(C-D)** Vacuolar changes in the large cells with eosinophilic cytoplasm, vesicular nucleus, prominent nucleolus and neuropil-like background. There are also focally clustered small cells of oligodendroglial character **(C)**. Scale bars: **A** = 200 μm; **B** = 2 mm; **C**-**D** = 50 μm.

Both cases showed multiple (numerous in Case 2) discrete, well-demarcated, focally coalescent nodules, which were found predominantly within the white matter and in the deep cortex traversing the white matter – cortical junction (Figure 3). The lesions were sharply demarcated and hypomyelinated on LFB/N although a few myelinated fibres were detected running through the lesion. There were abnormal neuronal cells with round nuclei, vesicular chromatin and a nucleolus and a moderate amount of eosinophilic, coarsely vacuolated cytoplasm. There was no nuclear atypia and no binucleate cells or ganglion cells were noted. The background had a delicate neuropil-like appearance and contained oligodendroglial-like cells, often in small clusters in Case 1 (Figure 3C), reactive astrocytes and microglial cells, also including a few foamy macrophages sometimes containing LFB/N positive myelin breakdown products. Fine extracellular vacuolation was also noted. There were no mitotic figures or vascular proliferation seen. The nodules were separated by unremarkable brain parenchyma. The adjacent cortex showed normal lamination. There was no evidence of storage material with PAS and Sudan black.

Case 2 showed typical histological features of MND in the brain and spinal cord.

### Immunocytochemistry

**Figure 4:**
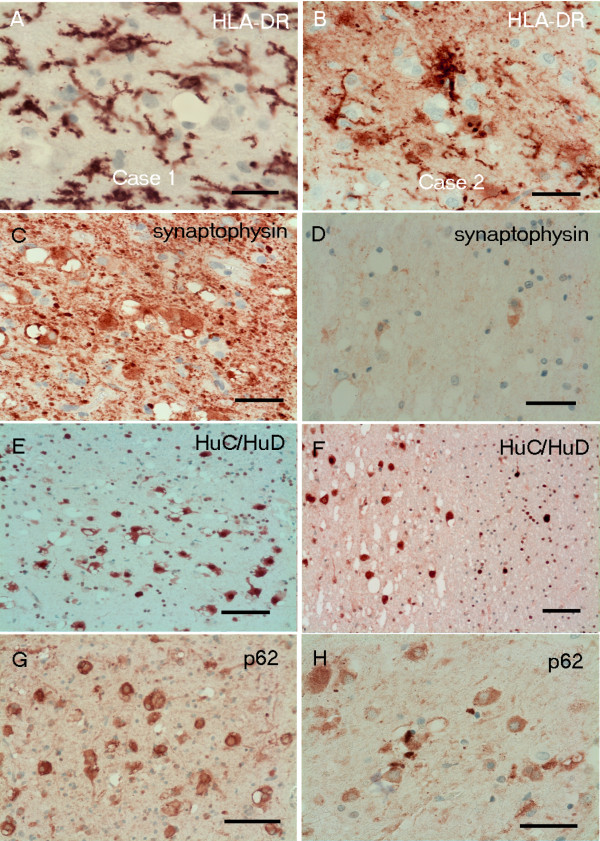
**Immunohistochemistry Case 1 (A,C,E,G) and Case 2 (B,D,F,H). (A-B)** There are HLA-DR positive reactive microglial cells but the vacuolated cells are negative. **(C-D)** The immunoreactivity with synaptophysin is strong in Case1 **(C)**, while the vacuolated cells are only moderately positive in Case 2 **(D)**, possibly due to overfixation. **(E-F)** There is strong HuC/HuD immunoreactivity in the vacuolated cells, as well as in a few round nuclei. **(G-H)** The p62 highlights the lesion by diffuse cytoplasmic labelling of the large vacuolated cells. Scale bars = 100 μm.

GFAP highlighted the reactive astrocytes within the nodules. There was marked microglial cell activation throughout the lesions as demonstrated by up-regulated HLA-DR and CD68 but the vacuolated cells were negative (Figure 4A-B). The vacuolated cells were immunoreactive for synaptophysin, while the fibrillary tumour matrix showed decreased synaptophysin expression compared to the adjacent cortex (Figure 4C). The intensity of staining was less in case 2, possibly due to prolonged fixation (Figure 4D). There was strong HuC/HuD immunoreactivity in the vacuolated cells and a few round nuclei were also labelled in the lesions as well as in the normal white matter (Figure 4E-F). Chromogranin, pan-NF and NF200 KD were negative in the neoplastic cells, but neurofilaments revealed positivity within axons running through the matrix of the nodules, although a few axons appeared to be disrupted. The vacuolated cells were intensely immunoreactive with p62 in both cases (Figure 4G-H). There was no immunoreactivity for nestin and CD34 within or around the nodules. The proliferation activity was less than 1% by Ki67, and was restricted to small nuclei, probably glial or microglial. The nuclei of the vacuolated cells did not show Ki67 immunoreactivity. Both cases were negative with IDH1 specific for R132H mutation.

**Figure 5:**
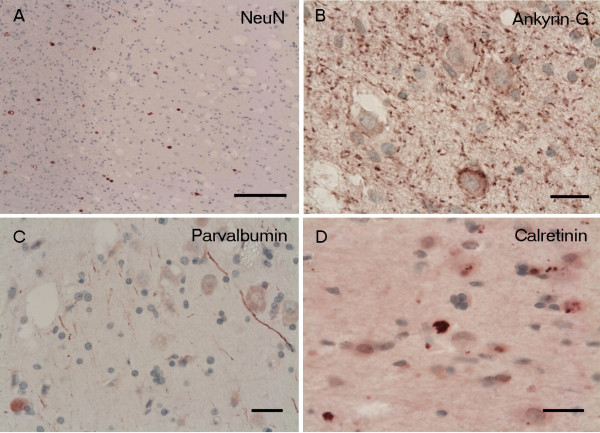
**Immunohistochemistry Case 1. (A)** The vacuolated large cells are negative with NeuN although scattered normal white matter neurons are still labelled. **(B)** Ankyrin-G antibody (marker of axon initial segment = AIS) shows somatic expression in the vacuolated large cells but no labelling of the AIS apart from scattered puncta in keeping with the nodes of Ranvier. **(C)** Parvalbumin reveals only weak staining in some of the vacuolated large cells, while calretinin **(D)** shows strong granular cytoplasmic expression. Scale bars: **A** = 200 μm; **B**-**D** = 50 μm.

Some antibodies did not work on the overfixed autopsy material; therefore additional immunohistochemistry was carried out on Case 1 only. NeuN was negative within the vacuolated cells (Figure 5A), but normal staining of neurons was seen in the overlying cortex with no disturbance of the laminar architecture. Ankyrin-G, a marker for the axon initial segment (AIS) [6], was normally preserved in the cortex but showed somatic immunoreactivity without axonal labelling of the neurons of the nodules (Figure 5B). There was also faint immunoreactivity with parvalbumin (Figure 5C) and calbindin strongly labelled some cytoplasmic granules of the neoplastic vacuolated cells (Figure 5D).

In Case 2, the immunophenotype confirmed the diagnosis of MND with limbic and mild neocortical TDP-43 pathology. Occasional TDP43 positive oligodendroglial inclusions were seen within the lesions, as frequently as in the rest of the white matter.

### Molecular genetic tests

There was no evidence of mutations of BRAF V600E, IDH1 and IDH2 in either case. MGMT promoter showed borderline methylation in Case 1, while Case 2 was unmethylated.

### Electron microscopy

**Figure 6:**
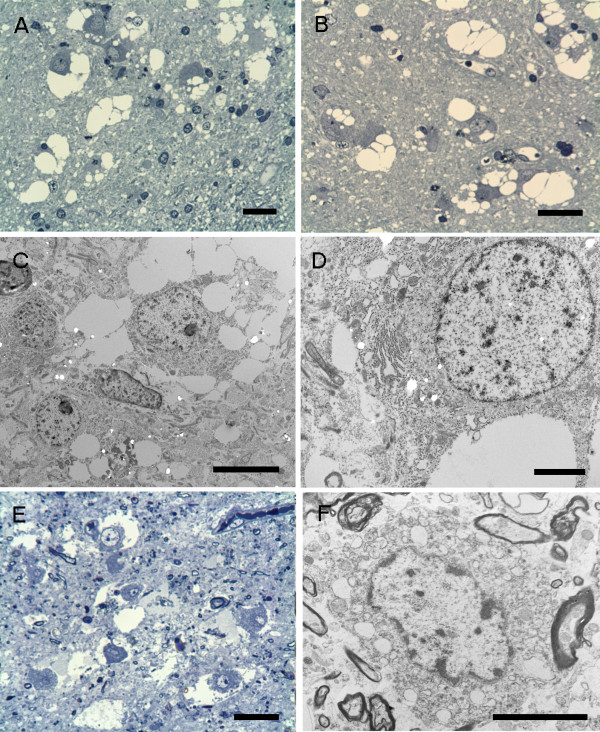
**Semithin sections and electron microscopy.** Case 1 **(A-D)**. **(A-B)** Conspicuous vacuolar changes in the neuronal cells and the neuropil-like background. **(A)** Mild accumulation of small round cells is noted at the edge of the nodule. **(C-D)** Electron microscopy reveals non-membrane bound vacuolation. **(D)** Small vacuolated areas within the cisterns of the endoplasmic reticulum are also noted in the neuron. Case 2 **(E-F)**. The vacuolar changes are similar those seen in Case 1 although the less well-preserved due to post-mortem changes. (**A**-**B**, **E** - semithin sections stained with Toluidine blue). Scale bars: **A**, **B**, **E** = 25 μm; **C** = 5 μm; **D**, **F** = 20 μm.

The preservation was better in Case 1. In both cases, the vacuolated cells showed ultrastructural features of mature neurons with round to oval frequently indented nuclei with nucleoli and cytoplasm which was rich in mitochondria and rough endoplasmic reticulum (Figure 6). In some cells dilated cisterns of endoplasmic reticulum were seen. There were lipofuscin granules, more readily seen in Case 2. Extensive non-membrane bound vacuolation was seen within the cytoplasm as multiple small vacuoles or coalesced into a large vacuole. Synapses were not found. Surrounding the neurons there were unmyelinated and small myelinated nerve fibres and glial cell processes. Some vacuolation was noted outside the neuronal perikaryon, possibly in the nerve fibres, but this could not be determined with certainty. There was no evidence of a storage disorder.

## Discussion

We have described two cases, a surgical resection and the other an autopsy specimen, with histological features similar to those recently described as MVNT by Huse et al. [2]. The radiological features were also similar, showing lesions with T2 signal hyperintensity and increased signal in FLAIR in the subcortical white matter; being subtle in Case 1 and extensively involving the posterior part of the temporal lobe in Case 2. Both our cases were favoured to be DNT by the radiologists as having absence of mass effect and no surrounding oedema [7]. Multinodularity was demonstrated both radiologically and by macroscopic examination in our cases. The discrete and coalescing grey nodules abutted on the deep cortex although most lesions were subcortical. This type of localisation may also occur in DNT but appears also to be characteristic of MVNT [2].

Until the neuronal nature of the strikingly vacuolated cells could be established, the uniform greyness of the lesions and the loss of myelin staining raised the differential diagnosis of demyelinating disease. In Case 2 the nodules were numerous but there were none in a periventricular location as usually observed in multiple sclerosis. There was prominent microglial activation with some foamy macrophages which sharply demarcated the abnormal areas from the surrounding white matter. Microglial marker upregulation has been found in tumours from patients with epilepsy, such as diffuse and nodular DNT, ganglioglioma and pleomorphic xanthoastrocytoma, and has also been described in focal cortical dysplasia (FCD) [8,9]. However, there was no perivascular lymphocytic inflammatory reaction and the vacuolated neurons were clearly negative with microglial/macrophage markers. Moreover, some myelinated axons were still noticeable within the nodules. It is possible that the vacuolation, affecting not only the abnormal neurons but the neuropil as well, may be responsible for the hypomyelination observed in the nodules. There was no indication of any metabolic disorder or ion imbalance in any of our cases to account for a non-inflammatory type of demyelination.

The vacuolated cells failed to express NeuN (available in Case 1). It appeared to label only the unaltered normal mature neurons of the white matter but not the vacuolated neurons. There was also no chromogranin or neurofilament positivity in the cytoplasm of those cells but axonal immunoreactivity with neurofilament was detected in the axons running through the lesion. We were able to confirm consistent labelling of the vacuolated cells by HuC/HuD as reported by Huse et al. [2]. In addition we also noted immunoreactivity in small round nuclei being present not only in the lesion but also in the normal white matter, suggesting labelling of some of the normal oligodendroglial cells. Previous immunohistochemistry studies with anti-HuC/HuD described positivity, in addition to small cell carcinoma of lung, in neoplasms of differentiated neuronal type and embryonal tumours with neuronogenic potential but no glial tumours apart from occasional oligodendrogliomas [10-12]. Immunolabelling of normal oligodendroglial cells has not been reported previously and this may be attributed to more aggressive antigen retrieval method used by us. We found strong synaptophysin expression in Case 1 but this was weaker in Case 2, probably due to prolonged fixation in autopsy material. The synaptophysin expression was variable in the series of Huse et al.; three cases were positive, three cases were negative and four cases had focal weak labelling. We also demonstrated increased somatic Ankyrin-G expression of the vacuolated neurons; however, the AIS was not labelled in neurons within the lesions. Changes in the structure of the AIS may result in modifications of the excitability of these neurons, as described previously [6,13]. We also found increased p62 immunoreactivity within the abnormal. Although the p62 labelling is not specific, defective autophagy may be suspected in the vacuolated neurons.

Electron microscopy confirmed the neuronal nature of the vacuolated cells and the presence of a neuropil, which also showed some vacuolation. The cause of the vacuolation of neurons could not be established nor could it be related to a specific cell organelle. There was no accumulated material. A selective artefact affecting the neurons within the lesion is unlikely.

The question remains unanswered as to whether the nodules of markedly vacuolated neurons represent definite neoplastic transformation or aberrant development. Nodular heterotopia has similar naked eye appearances although the nodules are usually more well demarcated from the cortex and do not show coalescence [14,15]. However, vacuolation is not a feature of heterotopia as they tend to show normal composition of neurons and neuropil without significant reactive astrocytic or microglial reaction [14,15]. Some nodular heterotopias are associated with cortical dysplasia in the overlying cortex [15], but there was no cortical dysplasia observed in our cases. The vacuolated neurons failed to express NeuN which is preserved in heterotopic neurons and there was abnormal immunomarker expression in the vacuolated neurons compared to normal cortical neurons [14,15]. Reactive astrocytosis and microglial cell activation, which was prominent within the lesions in both our cases, also not a feature of nodular heterotopias. The reactive changes can be explained as the consequence of chronic seizures in Case 1, but there were no seizures in Case 2. The presence of some foamy macrophages within the nodules, a few with myelin debris, is also suggestive of an acquired lesion.

Most importantly, the differential diagnosis includes the well known glioneuronal neoplastic lesions. We saw no ganglion cell morphology or atypia characteristic of ganglioglioma and gangliocytoma. Although vacuolar alteration is not uncommon in ganglion cell tumours, those are rarely and inconsistently seen in the ganglion cell component, and more likely to manifest as microcystic or myxoid change. There was also no typical accompanying glial component in these lesions, such as Rosenthal fibres, eosinophilic granular bodies, microcalcification or lymphocytic inflammatory reaction [16-18]. The lack of neurofilament and chromogranin immunoreactivity would also speak against ganglioglioma. Huse et al. described focal immunoexpression of CD34 in 7 out of 10 cases in ramified processes of small cells but no immunoreactivity in the vacuolated neurons. Such CD34 immunoreactive cells have been found in various epileptogenic lesions, including focal cortical dysplasia type IIB, and frequently colonize the cerebral cortex adjoining gangliogliomas, pleomorphic xanthoastrocytomas, observed in association with complex and diffuse type of DNT and even detected in high grade gliomas [8,19,20]. We did not observe CD34 immunoreactivity in our two cases.

Molecular genetic testing showed no mutation of BRAF V600E, which may be found up to 50% of gangliogliomas and pleomorphic xanthoastrocytomas [21], in either of our 2 cases or in 4 cases tested by Huse et al. Nevertheless, Huse et al. demonstrated a point mutation involving MEK 1 (MAP 2 K1) in 1 out of 8 tested cases.

The presence of an oligodendroglial/OLC component is suspected in this lesion. Huse et al. described OLIG2 expression in all of their lesions [2]. We also detected increased a number of oligodendroglial-like cells and oligodendrocytes within and sometimes at the border of the nodules. The OLC is a characteristic cell component of DNT and is essential for the diagnosis [22,23]. There was no evidence of so-called “specific glioneuronal lesion” in our cases, but this is absent in the diffuse DNT, and may not be seen in the complex multinodular DNT [8,24]. However, the predominantly neuronal composition of these lesions and the striking cytoplasmic vacuolation has not been reported so far in DNT.

MVNT was reported as a lesion associated with adult onset seizures. Of the ten cases reported by Huse et al., five cases had adult onset epilepsy, most with a short history of seizures or episodic confusion (between 1 to 5 years) [2]. Three patients had only a single episode and one had a possible episode, which prompted neuroimaging and the discovery of the lesions. The last, a 54 year-old man, presented with seizures for over 40 years starting in childhood. The latter is similar to our Case 1 (seizure onset at 8, epilepsy for 28 years). Our second case, on the other hand, an elderly patient with MND and no history of seizures had extensive temporal lobe lesions which were discovered only by routine MRI scanning as per the MND protocol. While it is possible that mild symptoms went unnoticed in the face of a severe neurological illness, it is likely that other non-seizure associated incidental cases may come to light in the future.

The biological behaviour of MVNT appears to be benign, similar to DNT. Regardless of the extent of excision, no tumour recurrence was noted in the 8 cases available for follow up in the series of Huse et al. [2]. Our cases also did not show re-growth or increase in size of the lesion on follow-up images. Only one out 8 patients of Huse et al. remained symptomatic after subtotal lesion removal, suffering seizures and cognitive disturbances. Although the frequency of seizures decreased after surgical resection (Case 1), our patient did not become seizure-free.

## Conclusions

Many of the tumour-like glioneuronal lesions associated with chronic epilepsy appear to be clearly neoplastic, others continue to confound, straddling the boundary between malformation and neoplasm. The ten MVNT cases recently reported by Huse et al. were named as tumour but share features with malformation. On the other hand, this type of lesion shows similarities at least in behaviour with DNT and it is possible that the MVNT may even form part of the spectrum of DNT. MVNT may also present as an incidental lesion without seizures.

## Consent

Written informed consent for publication were obtained for case report using accompanying MRI and histological images without identifiable patient details.

## Competing interests

The authors declare they have no competing interests.

## Authors’ contributions

IB, OC, SAS and MH made the pathological diagnosis. RS performed the surgery and RE performed the electrophysiology. Molecular genetic testing was performed by RL. IB and MH drafted the manuscript. All authors contributed to the manuscript and approved the final manuscript.
